# Arachidonic acid metabolism is elevated in *Mycoplasma gallisepticum* and *Escherichia coli* co-infection and induces LTC4 in serum as the biomarker for detecting poultry respiratory disease

**DOI:** 10.1080/21505594.2020.1772653

**Published:** 2020-06-03

**Authors:** Zhiyong Wu, Chunli Chen, Qiaomei Zhang, Jiaxin Bao, Qianqian Fan, Rui Li, Muhammad Ishfaq, Jichang Li

**Affiliations:** a College of Veterinary Medicine, Northeast Agricultural University, Harbin, P. R. China; b Heilongjiang Key Laboratory for Animal Disease Control and Pharmaceutical Development, Northeast Agricultural University, Harbin, P. R. China

**Keywords:** Multiple respiratory diseases, metabolomics, arachidonic acid, leukotriene C4, biomarker

## Abstract

Outbreaks of multiple respiratory diseases with high morbidity and mortality have been frequently reported in poultry industry. Metabolic profiling has showed widespread usage in metabolic and infectious disease for identifying biomarkers and understanding of complex mechanisms. In this study, the non-targeted metabolomics were used on *Mycoplasma gallisepticum* (*MG*) and *Escherichia coli* (*E.coli*) co-infection model in serum, which showed that Leukotriene C4 (LTC4), Leukotriene D4 (LTD4), Chenodeoxycholate, Linoleate and numerous energy metabolites were varied significantly. KEGG enrichment analysis revealed that the metabolic pathways of linoleic acid, taurine and arachidonic acid (AA) were upregulated. To further characterize the consequences of co-infection, we performed an AA metabolic network pathway with metabolic products and enzyme genes. The results showed that the expression of LTC4 increased extremely significant and accompanied with different degree of infection. Meanwhile, the AA network performed the changes and differences of various metabolites in the pathway when multiple respiratory diseases occurred. Taken together, co-infection induces distinct alterations in the serum metabolome owing to the activation of AA metabolism. Furthermore, LTC4 in serum could be used as the biomarker for detecting poultry respiratory disease.

**Abbreviations:**

MG: *Mycoplasma gallisepticum;* E.coli: *Escherichia coli*; AA: Arachidonic acid; LTC4: Leukotriene C4; CRD: chronic respiratory diseases; KEGG: Kyoto Encyclopedia of Genes and Genomes; LTs: leukotrienes; PGs: prostaglandins; NO: nitric oxide; HIS: histamine; PCA: Principal Component Analysis; PLS-DA: Partial Least Squares Discriminant Analysis; CCU: color change unit; UPLC: ultra-performance liquid chromatography; MS: mass spectrometry; DEMs: differentially expressed metabolites; ELISA: enzyme-linked immunosorbent assay; SD: standard deviation; VIP: Variable importance in the projection

## Introduction

The primary pathogens of respiratory disease in poultry include *Mycoplasma gallisepticum* (*MG), Escherichia coli* (*E.coli), Avian influenza virus* and *Infectious bronchitis virus*, which have caused huge economic losses to the poultry industry worldwide [1–3]. Recently, outbreaks of multiple respiratory diseases with high morbidity and mortality have been frequently reported `^4^, ^5^]. Due to the intensification of commercial poultry production, the explosive multiple respiratory infections has become an urgent problem. However, the in-depth studies on multiple infections are still difficult to conduct because of the complexities of co-infection.

*MG*, the smallest pathogen that primarily causes chronic respiratory diseases (CRD) in poultry birds [6,7]. *MG* is often associated with co-infection outbreaks of other pathogens, which may due to a down-regulation of the host immune response by *MG* infection [8]. *Colibacillosis* infection caused by avian pathogenic *E.coli* (APEC) is also an economically important bacterial disease in poultry [9]. Respiratory diseases may be induced by various viral and bacterial agents, either alone or in combination [10]. Especially, *MG* usually infects birds along with co-infection of *E.coli* and viral pathogens [11]. It is therefore important to explore the mechanism of co-infection and conduct targeted drug therapy.

Although the multiple respiratory diseases are very complex in terms of pathogenesis and the relationship between inflammation, clinical disease and response to treatment, with significant improvements in analytics platforms and the reduction in costs, the mechanism for using ’omics to elucidate disease has grown exponentially in recent years [12,13]. A previous study reported the metabolite profiling combined with bioinformatics of *MG* mutants which could reveal the likely functions of virulence associated genes [14]. In addition, the application of copolymerization and canonical correlation analysis also identified a number of important conditional correlations between metabolites and transcripts of *E.coli* [15]. Moreover, Dai found the biomarkers of iron metabolism which facilitate clinical diagnosis in *Mycobacterium* infection [16]. In line with the development of ’omics, the discovery of biomarkers has greatly advanced the development of disease diagnosis and drug targeted therapy.

Breathing difficulties caused by airway inflammation in respiratory diseases are the main cause of poultry death, and the common substances which induce contraction of tracheal smooth muscle are mainly leukotriene (LT), prostaglandins (PG), nitric oxide (NO), histamine (HIS), etc [17–19]. NO was used to assess the underlying mechanisms of airway and lung inflammation for investigating asthma [20]. We previously demonstrated that co-infection of *MG* and *E.coli* triggers inflammatory injury involving IL-17 signaling pathway [21]. Furthermore, IL17A was reported that induce neutrophilic inflammation, airway hyperresponsiveness, steroid insensitivity and airway remodeling [22]. Previous studies have shown that co-infection with *MG* and *E.coli* could induce more severe inflammatory injury than individual infection. While, the role of metabolites in pathogenesis remains unclear.

There are no precedents for the screening of biomarkers or biochemical indicators in metabolomics for the study of poultry respiratory diseases. In this study, we conducted a non-targeted metabolomics to probe the metabolic changes in a co-infection (*MG* and *E.coli*) model. The purpose is to delve into the molecular mechanisms of co-infection and to identify specific biomarkers for poultry respiratory diseases. The emergence of biomarkers in the respiratory system can provide evidence for the pathological diagnosis of the poultry industry, and provide a more comprehensive basis for its prognosis and treatment options, thereby reducing unnecessary economic losses.

## Methods and materials

### Mycoplasma *strain and* E.coli

The strain R_low_ of *MG* was provided by Harbin Veterinary Research Institute (Chinese Academy of Agricultural Sciences, Harbin). The culture conditions and the detection of the density for *MG* were consistent with our previous study [23,24]. In short, modified Hayflicks medium containing 0.05% Penicillins, 0.1% Nicotinamide adenine dinucleotide, 10% freshly prepared yeast extract, 20% fetal bovine serum and 0.05% thallium acetate. *MG*, in its mid-exponential phase indicated by the color change of phenol red dye from red to orange, was used to challenge chickens at the density of 1 × 10^9^ CCU/ml (color change unit per milliliter) in the culture medium. The concentration of *E. coli* was adjusted to 10^9^ CFU/ml before infection.

## Experimental models establishment and grouping

Forty (1-day-old) White Leghorn chickens were purchased from Chia Chau Chicken Farm (Harbin, China) and were assigned randomly to four groups namely (A) Control group, (B) Co-infection group, (C) *MG* group, (D) *E.coli* group (10 chickens per group). The chickens were in healthy conditions, *MG* and *E.coli* (O78)-free and did not undergo vaccination and raised to the 7^th^ day in four separate environmentally controlled chambers. Meanwhile, the chickens were half male and female and each group was housed in a positive-pressure fiberglass isolator and provided with antibacterial-free balanced feed and fresh drinking water ad libitum. (A) Control group, Fed in the same environment and kept until the end of experiments. (C) *MG* group, the method of *MG* infection was constructed by left caudal thoracic air sac inoculation with *MG* R_low_ strain 0.2 mL (1 × 10^9^ CCU/ml) at 7^th^ day. (D) *E.coli* group, the *E.coli* infection model was injected at a dose of 0.1 ml *E.coli* (10^9^ CFU/ml) intraperitoneally at day 10. The Co-infection group (B): 0.2 ml of *MG* medium (1 × 10^9^ CCU/ml) was injected into the left caudal thoracic air sac at 7^th^ day, and 0.1 ml of *E.coli* bacteria (10^9^ CFU/ml) was injected intraperitoneally at day 10 [25]. Four methods were used to verify whether the three models were successfully established including PCR tests, serological tests, pathological observations and pathogen isolation as previously described [21,26].

## Sample collection

At 13^th^ day, 10 chickens from each group were humanely sacrificed to avoid pain and suffering of chickens. The lung and tracheal samples were collected from each groups for further experimental analyzes. RNA extracted by Trizol reagent (Invitrogen Inc., Carlsbad, CA) from lung tissue was utilized to construct the final library (BGISEQ-500 RNA-Seq Library) based on the manufacturer’s instructions. Library was validated on the Agilent Technologies 2100 bioanalyzer. GEO accession number is GSE130015.

Blood samples were collected from each group in a vacuum blood collection tube at 37°C for 1 h for solidification stratification. Then centrifuge at 3,000 rpm for 5 min, and take the supernatant to a clean centrifuge tube. Centrifuge at 12,000 rpm and 4°C for 10 min. The serum samples were collected and frozen at −80°C after liquid nitrogen quick freezing [27]. The present study was conducted under the approval of Laboratory Animal Ethics Committee of Northeast Agricultural University (Heilongjiang province, China) in accordance with Laboratory animal-Guideline for ethical review of animal welfare (GB/T 35,892–2018, National Standards of the People’s Republic of China).

## Non-targeted metabolomics study on serum of Control and Co-infection groups

16 serum samples of A and B groups were examined by the LC-MS system as follows. Briefly, an ACQUITY UPLC HSS T3 column (100 mm*2.1 mm, 1.8 μm, Waters, UK) was used for all chromatographic separations by an ultra-performance liquid chromatography (UPLC) system (Waters, UK). The column temperature was 50°C and the flow rate was 0.4 ml/min. The mobile phase of solvent A were water and 0.1% formic acid, and solvent B were methanol and 0.1% formic acid. The metabolites were eluted using the following gradients: 0～2 min, 100% phase A; 2 ~ 11 min, 0% to 100% B; 11 ~ 13 min, 100% B; 13～15 min, 0% to 100% A.

Mass spectrometry was performed on a high-resolution tandem mass spectrometer (MS) Xevo G2 XS QTOF (Waters, UK). The Q-TOF was operated in both positive and negative ion modes. The detailed parameters were as follows: the capillary voltages were set at 3.0 kV and 2.0 kV, respectively for positive and negative ion mode, the sampling cone voltages were set at 40.0 V. The MS data were acquired in Centroid MSE mode. The metabolomics in centroid mode range were set from 50 to 1200 Da and the scan time was 0.2 s. For the MS/MS detection, all precursors were fragmented using 20–40 eV, and the scan time was 0.2 s. The collected MS data was analyzed by using the commercial software Progenesis QI (version 2.2) (Waters, UK) and BGI’s (Beijing Genomics institute, China) metabolomics R software package metaX [28], and the metabolite identification was based on the database KEGG.

## Detection of biomarkers and biochemical indicators by ELISA

Six samples (serum, lung and trachea, respectively) from each groups were prepared for enzyme-linked immunosorbent assay (ELISA) kits in accordance with the manufacturer’s instructions (Kenuodi Biotechnology Co., Ltd. Fujian, China.). NO assay kit was purchased from Nanjing Jiancheng Bioengineering Institute (Nanjing, China). Four biomarkers and biochemical indicators activities were detected including LTC4, LTD4, HIS, NO. The lung and trachea tissue samples were prepared as mentioned previously [29].

## Statistical analysis

Data are presented as mean results ± standard deviation (SD). The significance was determined using one-way ANOVA followed by Dunnett’s T3 test and unpaired t test (parametric test). The data were analyzed by using the GraphPad Prism (version 5.01). The correlation network of potential metabolites was made by Metscape (MetScape v3.1.3) [30]. The box and bubble plots were made by MetaboAnalyst (MetaboAnalyst v4.0) [31].

## Results

### Arachidonic acid metabolism is activated in serum from Co-infection group

**Figure 1.**
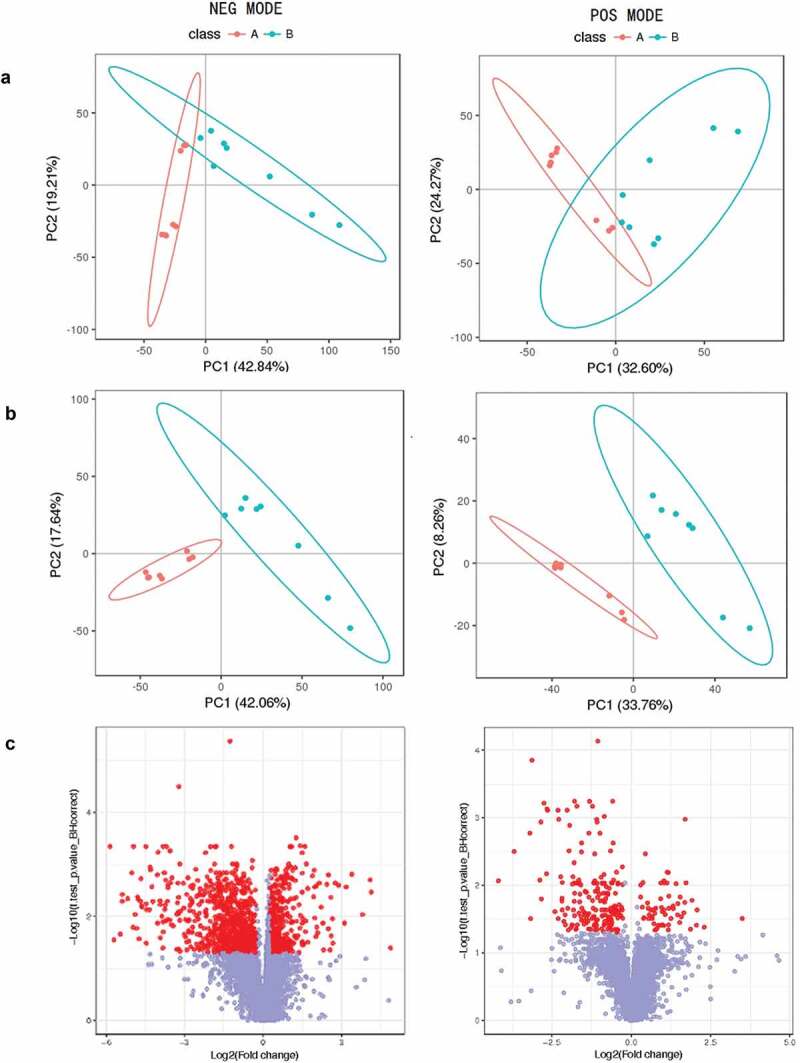
(a). PCA scores plots, (b). OPLS-DA scores plots, (c). VIP-plot of OPLS-DA for serum samples of the co-infection group (blue) versus healthy controls (red) in negative and positive ion mode (label 1, 2). Log2 (fold change) is the abscissa, and the negative logarithm of q-value is the ordinate in (C). The red points represent fold change ≥ 1.2 or ≤ 0.8333 and q-value < 0.05, and the remaining points are gray.

**Figure 2.**
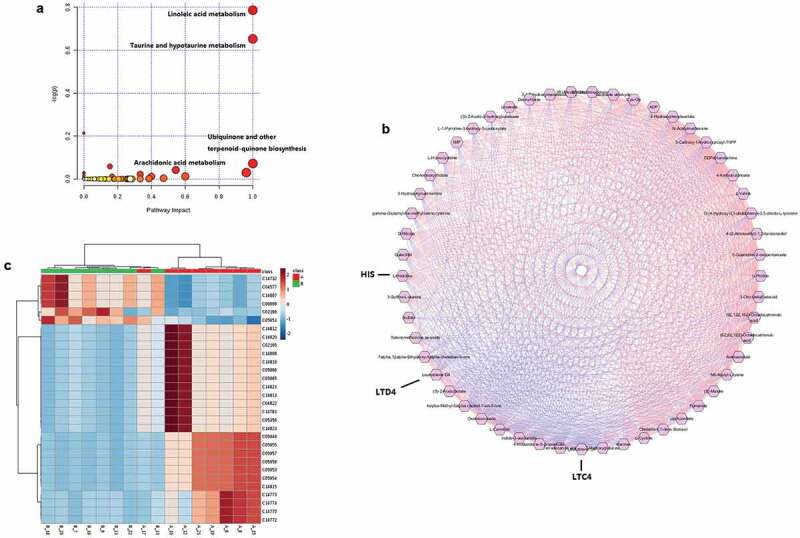
(a). Bubble plot of pathway analysis with MetaboAnalyst of potential metabolites in serum. (b). A correlation network of potential metabolites analysis with Metscape in serum. The regular hexagon indicate potential biomarkers, blue lines represent positive correlation, and red lines represent negative correlation. (c). The heat map of metabolites related to the arachidonic acid metabolic pathway.

The serum samples were separated and collected by UPLC-QTOF/MS using positive and negative modes. The PCA (principal component analysis) and PLS-DA (partial least-squares discriminant analysis) of metabolites indicated that co-infection had a systemic metabolic profile different from that of control group (Figure 1a,b). Differentially expressed metabolites (DEMs) were screened in combination with univariate analysis of fold change and q-value. Screening conditions: 1) VIP ≥ 1; 2) fold change ≥ 1.2 or ≤ 0.8333; 3) q-value < 0.05. The intersection of these three conditions was obtained, and the cross ones were the DEMs (Totally 928 metabolites, including positive and negative modes). The results of the differential metabolites are presented as a Volcano plot (Figure 1.C). The metabolic pathways of the linoleic acid (3/4 metabolites), taurine and hypotaurine (4/6 metabolites), AA (14/31 metabolites) were activated significantly, which were found using MetaboAnalyst analysis (Figure 2.A). The complete data of pathways was recorded in Supplementary material 1.

## Correlation analysis of DEMs

The information of differential metabolites was substituted into Metscape for correlation analysis. Correlation calculator in combination with Metscape can also be used to discover the connectivity between metabolites where pathway information is not readily available. A correlation network of potential metabolites related to effects of co-infection was exhibited in Figure 2.B. Totally, 53 potential metabolites and the correlation table were found and shown in Supplementary material 2 and 3, including O-(4-Hydroxy-3, 5-diidophenyl)-3, 5-diiodo-L-tyrosine, Leukotriene C4, Leukotriene D4, Chenodeoxycholate, Linoleate and numerous energy metabolites. Correlation analysis of potential biomarkers and biochemical indicators might be valuable for understanding the pathological process of co-infection. According to the results of two metabolomics analysis methods, we selected the AA metabolic pathway and LTC4 for further studies.

## Changes in expression of arachidonic acid pathway-related products and genes

**Figure 3.**
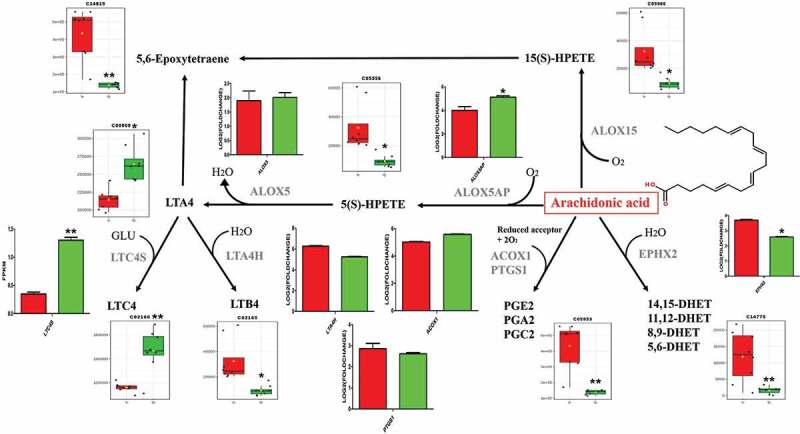
7 genes and 13 metabolites in the co-infection group metabolic signature mapped onto the arachidonic acid pathway (Arachidonic acid structure as shown in it). Relative expressions are shown as box (metabolites) and column (genes) plots, red for control group and green for co-infection group. The ordinate of the box plot is the ionic strength, and the ordinate of the column plot is Log2 (fold change) or FPKM (gene expression). Bars represent the mean ± SD. The values with star differ significantly (with “*”, 0.01 < P < 0.05) or very significantly (with “**”, P < 0.01) between Group A and B.

The metabolites heat-map is shown in Figure 2.C. Based on the expression of AA pathway products and the pathway information corresponding to the KEGG, we have drawn a complete AA pathway map with changes in expression, as shown in Figure 3. 14/31 metabolites in the co-infection-specific metabolic signature mapped onto their interconnecting pathways and the involved genes were also added to the map. The expression of related genes was screened from our previous RNA-seq results by the BGI data mining online website (http://report.bgi.com) as explained in previously [21]. The results showed that the expression of LTC4 S and ALOX5AP in co-infection was elevated significantly compared to control group, while the expression of CYP2C21 L and LTA4 H were decreased significantly, and the other genes showed no significant differences. ALOX15 was not expressed in each group. Among the expression of metabolites, LTA4 and LTC4 were elevated significantly, and other metabolites decreased significantly.

## The expression of LTC4, LTD4, HIS and NO in serum, lung and tracheal tissues by ELISA

**Figure 4.**
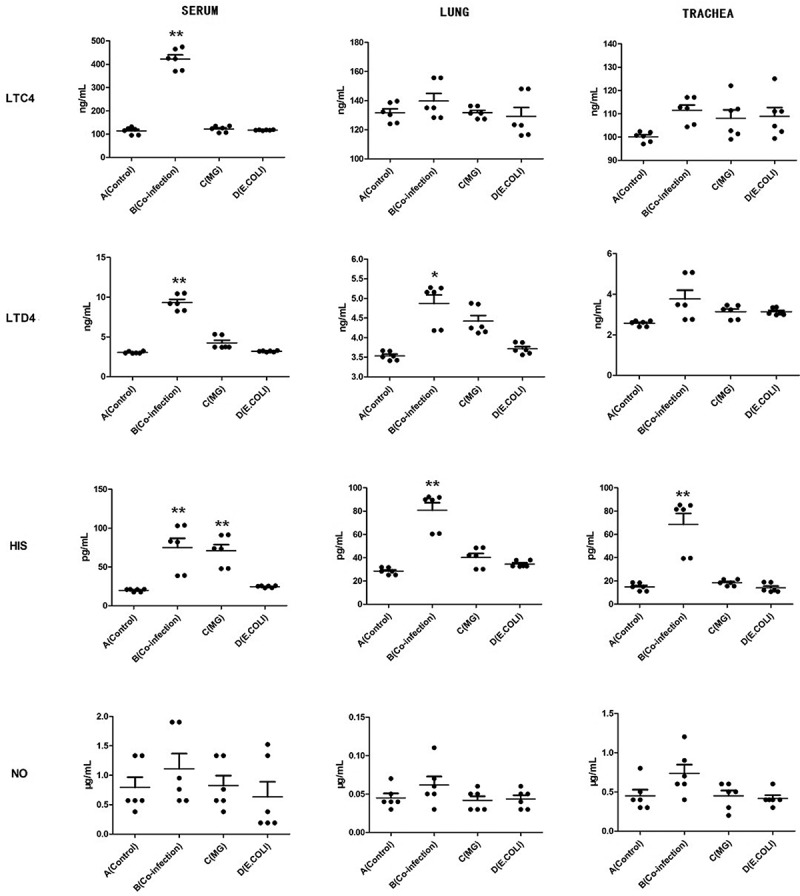
LTC4, LTD4, HIS, NO were detected by ELISA in serum, lung and tracheal tissues and performed with scatter plot. Bars represent the mean ± SD. The values with star differ significantly (with “*”, 0.01 < P < 0.05) or very significantly (with “**”, P < 0.01) between Group A and B.

To determine the biomarker of poultry respiratory disease, the concentrations of LTC4, LTD4, HIS and NO in the serum, lung and tracheal tissues were measured by using a sandwich ELISA. As shown in Figure 4, the expression of LTC4 and LTD4 were elevated extremely significant in the serum of co-infection group, while in the lung and tracheal samples showed no significant difference compared to other groups. The results of HIS indicated that the expression of co-infection group and *MG* group was higher than the other two groups in serum. Meanwhile in lung and tracheal tissues, the expression increased significantly only in the co-infection group Figure 4. (HIS). As shown in Figure 4. (NO), the expression of NO showed that there were no significant difference in any groups or tissues.

## Discussion

In addition to the basis of our previous study of co-infection model, the non-targeted metabolomics was carried out to analyze infection mechanisms and biomarkers in-depth. Initially, Principal Component Analysis (PCA) and Partial Least Squares Discriminant Analysis (PLS-DA) were performed on the obtained data. PCA is a multivariate statistical analysis method that transforms multiple variables into a few important variables (principal components) by dimensionally reduction techniques [32]. The PLS-DA analysis method performs partial least squares transformation PLS on the data, and then performs linear discriminant analysis LDA. PLS-DA is a multivariate statistical analysis method widely used by metabolomics [33]. Next, the differentially expressed metabolites were screened using the VIP values of the first two principal components of the multivariate PLS-DA model in combination with univariate analysis of fold-change and q-value. Totally 928 metabolites (remove duplicates) were screened in this study, including positive (245) and negative (882) modes. Finally, we sought to combine the DEMs with biochemical indicators to provide a more global characterization of the metabolic consequences of multiple respiratory infections.

After signal path enrichment analysis, we found that the DEMs were significantly enriched in the metabolic pathways of the linoleic acid (3/4 metabolites), taurine and hypotaurine (4/6 metabolites), AA (14/31 metabolites). These pathways are important for anti-inflammatory and immune responses during infection [34,35]. Studies have shown that pathway impact greater than 0.1 means that the pathway has enriched, and greater than 0.4 means significant enrichment [36]. In our study, the pathway impact of AA was 0.54. Furthermore, the AA metabolic pathway is more closely linked to others and has the most diverse metabolite enrichment, so we delved into it. AA is a polyunsaturated fatty acid that is abundantly present in the phospholipids of cell membranes, which induces prostaglandins and leukotrienes, participating in the afferent and efferent limbs of the immune system during host defense and inflammatory responses [37]. In our co-infection model, the AA pathway was also activated obviously, but the specific changes need to be further clarified.

The previous studies showed that AA produces four metabolic products including leukotrienes (LTs), prostaglandins (PGs), the 15-lipoxygenase enzymes and the cytochrome p450 enzymes [38]. In this study, the data from non-targeted metabolomics was combined with the results of RNA-seq to map onto the AA metabolic network pathways according to the MAP00590 on KEGG. The results of the metabolites and related enzyme genes involved in the AA metabolic pathway indicated that the complete AA network visualized the changes in the AA pathway after co-infection. LTC4 was significantly increased by the action of the relevant enzymes. In contrast, other products such as PGs showed significant down-regulation or no significant difference. The previous research showed that the severity of eosinophilic inflammation correlated directly with LTC4, LTD4, and LTE4 concentrations and inversely with PGE 2 concentrations [39]. The significant difference between LTs and PGs provides direction for further research, which lead us to speculate that severe inflammation might form a pivotal condition for co-infection. Our results reflected the significance of LTs in the AA pathway, which may induce LTC4 as the biomarker for diagnosis or treatment.

The correlation analysis of DEMs also induced LTC4, LTD4, HIS etc. as the potential biomarker for detecting multiple respiratory disease infections. Researches showed that LTC4, LTD4, HIS and NO play a physiological role in controlling bronchial airway reactivity [40,41]. These biochemical indicators can be used as a key factor in the diagnosis of disease course. Therefore, in this study, we aim to identify specific biochemical indicators of respiratory diseases in poultry. The expression of LTC4 and LTD4 were elevated extremely significant in the serum of co-infection group, and the expression of LTC4 was significantly higher than LTD4. In addition, the results of HIS indicated that the expression of co-infection group and MG group was higher than the other two groups in serum, which may be due to histamine being released faster than leukotriene or the course of the disease [42]. According to the three infection models, the degree of respiratory infection was judged from histopathological results [21]. Our results showed that LTC4 in serum showed significant differences in the degree of infection, which could support rapid diagnosis and prognosis treatment for poultry farms.

With the development of high-throughput sequencing, the application of metabolomics has made breakthrough in various fields. The ’omics drive the rapid accumulation of quantitative data and knowledge of the molecular networks of disease, which increase the development and use of quantitative disease models to facilitate efficient and safe drug discovery [43]. Metabolomics analysis of the response of metabolites can understand the developmental process of the disease from the root cause, and then propose more optimized solutions. Through the analysis of the metabolic network, biomarkers related to the disease can be found, which is of great significance for the clinical diagnosis of the disease [44,45]. Hence, we explored the potential of LTC4 as a biomarker of co-infection and discuss how activating AA metabolism might extend inflammation.

In conclusion, our metabolic results demonstrate that AA metabolism is activated in co-infection of *MG* and *E.coli*. Furthermore, correlation analysis induce LTs and HIS as the potential biomarker. The AA network constituted by the related metabolites and genes expressions showed that the LTs pathway is directly proportional, and the PGs is inversely proportional to infection. It is recommended that LTC4 in serum acts as a biomarker for detecting poultry respiratory diseases. This study uses metabolomics to provide new insights into multiple infections and provides new biomarkers for diagnosis and treatment.

## Supplementary Material

Supplemental Material
